# Spatial Analysis of the Relationship between Mortality from Cardiovascular and Cerebrovascular Disease and Drinking Water Hardness

**DOI:** 10.1289/ehp.6737

**Published:** 2004-04-15

**Authors:** Juan Ferrándiz, Juan J. Abellán, Virgilio Gómez-Rubio, Antonio López-Quílez, Pilar Sanmartín, Carlos Abellán, Miguel A. Martínez-Beneito, Inmaculada Melchor, Hermelinda Vanaclocha, Óscar Zurriaga, Ferrán Ballester, José M. Gil, Santiago Pérez-Hoyos, Ricardo Ocaña

**Affiliations:** ^1^Departamento d’Estadística i Investigació Operativa, Universitat de València, Valencia, Spain; ^2^Instituto Valenciano de Estadística, Generalitat Valenciana, Valencia, Spain; ^3^Departamento de Matemática Aplicada y Estadística, Universidad Politécnica de Cartagena, Cartagena, Spain; ^4^Departamento de Epidemiología, Dirección General de Salud Pública, Generalitat Valenciana, Valencia, Spain; ^5^Unidad de Epidemiología y Estadística, Escuela Valenciana de Estudios para la Salud, Generalitat Valenciana, Valencia, Spain; ^6^Escuela Andaluza de Salud Pública, Consejería de Salud, Junta de Andalucía, Granada, Spain

**Keywords:** environmental epidemiology, geographic information systems, hierarchical spatial models, relative risk, spatial smoothing

## Abstract

Previously published scientific papers have reported a negative correlation between drinking water hardness and cardiovascular mortality. Some ecologic and case–control studies suggest the protective effect of calcium and magnesium concentration in drinking water. In this article we present an analysis of this protective relationship in 538 municipalities of Comunidad Valenciana (Spain) from 1991–1998. We used the Spanish version of the Rapid Inquiry Facility (RIF) developed under the European Environment and Health Information System (EUROHEIS) research project. The strategy of analysis used in our study conforms to the exploratory nature of the RIF that is used as a tool to obtain quick and flexible insight into epidemiologic surveillance problems. This article describes the use of the RIF to explore possible associations between disease indicators and environmental factors. We used exposure analysis to assess the effect of both protective factors—calcium and magnesium—on mortality from cerebrovascular (ICD-9 430–438) and ischemic heart (ICD-9 410–414) diseases. This study provides statistical evidence of the relationship between mortality from cardiovascular diseases and hardness of drinking water. This relationship is stronger in cerebrovascular disease than in ischemic heart disease, is more pronounced for women than for men, and is more apparent with magnesium than with calcium concentration levels. Nevertheless, the protective nature of these two factors is not clearly established. Our results suggest the possibility of protectiveness but cannot be claimed as conclusive. The weak effects of these covariates make it difficult to separate them from the influence of socioeconomic and environmental factors. We have also performed disease mapping of standardized mortality ratios to detect clusters of municipalities with high risk. Further standardization by levels of calcium and magnesium in drinking water shows changes in the maps when we remove the effect of these covariates.

Cardiovascular disease is the primary cause of mortality in developed countries, with the exception of Japan. In Comunidad Valenciana, an autonomous region in Spain with approximately 4 million inhabitants, cardiovascular disease accounts for 35% of total female mortality and 46% of total male mortality (Melchor et al. 1998). Geographic variability within Comunidad Valenciana has been documented (Ferrándiz et al. 2000, 2002; Nolasco et al. 1992).

Previously published scientific articles have reported a negative correlation between drinking water hardness and cardiovascular mortality. Some results obtained in ecologic studies (Cradford et al. 1971; Lacey and Shaper 1984; Pocock et al. 1980) suggest that high levels of drinking water hardness (i.e., high concentrations of calcium and magnesium) are protective against cardiovascular diseases, mainly against ischemic heart disease.

Several surveys based on individual cases (Hall and Jungner 1993; Van der Vijver et al. 1992) have not confirmed the protective effect of calcium. Nevertheless, Rylander et al. (1991) and Yang (1998) suggest the beneficial effect of magnesium against coronary heart disease mortality as well as against cerebrovascular mortality. The results of these studies, obtained at the aggregated level, have been partially corroborated by case–control studies (Rubenowitz et al. 1996, 2000). These case–control studies, conducted in 18 southern municipalities in Sweden, found a protective effect of magnesium against acute myocardial infarction mortality but failed to find this effect for the total incidence in men.

A recent report by Marx and Neutra (1997) on the relationship between ischemic disease and magnesium in drinking water presented an analysis of several ecologic studies showing contradictory results, perhaps because the studies were not sufficiently specific to find the associations the investigators were exploring. The authors concluded their report by recommending that further studies be conducted to evaluate the apparent benefit of drinking water with high magnesium concentration.

A key issue to be addressed is the hypothetical temporal sequence between exposure and adverse health effects. It is unclear when to measure these factors, as there is no clear latency period. Some authors (Rubenowitz et al. 1996) have indicated that 1 year is sufficient to produce observable magnesium effects. However, other authors pointed out that longer periods of observations are needed (Marx and Neutra 1997).

More recently, Ferrandiz et al. (2003) studied the relationship between cerebrovascular mortality and calcium and magnesium concentrations in drinking water in 262 municipalities of the Valencia province in Spain from 1990 to 1995. They found a decreasing temporal trend, suggesting the cumulative effect of this beneficial factor, although this assertion needs further research.

Our research extends this last study, taking advantage of the Spanish Rapid Inquiry Facility (RIF). The RIF is an analytical tool for quick assessment that is applied to the data gathered in an information system developed within the European Health and Environment Information System (EUROHEIS) project and allows exposure analysis with covariates (Gómez et al. 2002).

First, we enlarged the period studied to 1991–1998. Second, we included all municipalities of Comunidad Valenciana, not just those belonging to the province of Valencia, thus enlarging our study from 263 to 538 municipalities. This provides a wider range of values for the factors being studied. Third, we considered ischemic [ICD-9 410-414 (World Health Organization 1978)] as well as cerebrovascular [ICD-9 430–438; (World Health Organization 1978)] diseases. Finally, data on drinking water hardness have also been completed and updated as a result of efforts to build a comprehensive environmental database inside the RIF.

## Material and Methods

### Data

One of the primary advantages of the RIF is the comprehensive database built into it. Mortality/morbidity, demographic, socioeconomic, and environmental data are assembled in a georeferenced system, allowing geographic representations of these phenomena. All data used in our study came from this source.

When constructing and updating the RIF database, mortality counts were obtained from the mortality registry of the Dirección General de Salud Pública. These numbers correspond to residents of Comunidad Valenciana and include deaths of those residents that occurred in or out of the region.

Similarly, environmental data such as those on the quality of drinking water were obtained from the Servicio de Calidad de Aguas at the Conselleria de Medio Ambiente. This agency has been analyzing public water supplies on an annual basis since 1989, although the frequency of the measurements varies between municipalities and some data are still missing. The average number of measurements in each municipality was 5.6 from 1991 to 1998. Fitting these data into the RIF database required statistical imputation of their values. Bayesian analysis of spatiotemporal models was used to perform this task as described in Abellán et al. (2003). Nevertheless, calcium and magnesium concentrations in drinking water were stable during the study, thereby minimizing the influence of the imputation methodology on the results of the analysis. Any missing value at a location was estimated by the average of the nearest 5 years at the same location. This simple procedure was sufficient in previous exploratory analyses.

Finally, demographic and socioeconomic data were provided by the Valencian Statistical Institute, where municipal statistics are updated regularly.

### Exposure Analysis

We performed exposure analysis within the RIF by defining regions (bands) composed of geographical units sharing similar levels of the risk factor under study.

These degrees of risk can be based on distance to a putative origin of risk (as in point source analysis) or on values of some environmental variables, as in our study. For each of the calcium and magnesium concentrations, we defined five bands, using as cut points the quintiles of their respective distributions on the 538 municipalities studied. To achieve uniqueness of these bands during the period studied, we used the values corresponding to 1991, the first year of our study. This choice was based, among other reasons, on a special program of water quality measurement used by the regional authorities that year. These regional authorities used a methodology common to all of the municipalities in Comunidad Valenciana.

Table 1 shows the values of calcium and magnesium defining those bands, the number of municipalities in each, and the percentage of total population of the region. The unevenness shown by these values is due to multiple ties in some of the cut points as well as to the variability of population sizes of these 538 municipalities. Populated municipalities make appreciable contributions to the band where they are allocated.

### Assessing Effects of Risk Factors

We performed statistical analysis using the estimation of the relative risk of each band *i* by the corresponding standardized mortality ratio *SMR_i_* = *O_i_/E_i_* of observed (*O*) to expected (*E*) mortality counts. In the computation of the expected counts *E_i_*, standardization by age groups was performed separately for each sex, as well as by levels of a deprivation index based on three municipal indicators: the ratios of unemployed individuals, the proportion of illiterates among individuals > 10 years of age, and number of vehicles per individual inhabitants (Arias et al. 1993). This deprivation index has been incorporated in the RIF as a new field attached to each municipality register. As a comparison region for each municipality, deprivation index standardization uses the band of the covariate that it belongs to and not the whole region of study. Thus, the model incorporates and controls the fact that risk could not be the same at different levels of the covariate.

In our study we performed indirect intrinsic standardization, using the population of the whole region as the reference population for each of the periods studied. The standardization procedure implicitly assumes that the expected rate in each stratum of a region is equal to the product of the relative risk of the region and a common mortality rate of this stratum. This is called the proportionality assumption (Wakefield et al. 2000), which must be checked to obtain valid conclusions. We performed a linear fit to the strata-specific rates of each municipality, and we did not observe clear departures from the linear assumption (Ferrándiz et al. 2003).

The output provided by the RIF includes the SMRs and their 95% confidence intervals in each band for every sex group.

Significant relative risks, that is, those for which confidence intervals do not include the value 1, are highlighted in the RIF output to facilitate their detection by visual inspection. Thus, we can identify those levels of the studied factors that correspond to unusually high or low relative risks.

Relying on this band-by-band inspection to identify the influence of a risk factor as significant has a statistical drawback. Because we are performing multiple tests to obtain a unified conclusion, we encounter the problem of simultaneous inference; that is, we risk identifying the global effect of an irrelevant factor as significant with a probability much higher than the nominal 5% level of each test. Thus, we have to protect against this global type I error by increasing the confidence level of our intervals or by performing a global test of homogeneity of bands before accepting any individual significant result.

This second alternative seems easier from the output of the RIF. It provides the observed *O_i_* and expected *E_i_* counts so that we can perform a χ^2^test of homogeneity of the number of (*n*) bands by computing the statistic


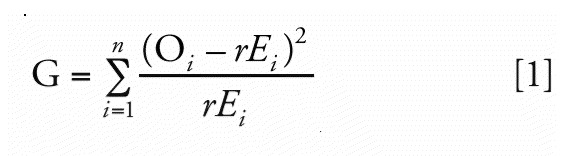


to be compared with the quantiles of the χ^2^ distribution with *n* – 1 degrees of freedom. In Equation 1, *r* is the ratio of total observed to total expected cases in the entire region, the maximum likelihood estimator of the common relative risk under the assumption of homogeneity of bands and Poisson-distributed counts.

### Handling Multiple Covariates

Standardizing mortality/morbidity rates by levels of a covariate as we have with age groups and deprivation index is a way of filtering its influence to allow the resulting statistics to be free from its effects. The remaining variability, if any, will be due to sources other than this covariate.

Covariate analysis, an option available within the RIF environment, performs this task. Once we have stipulated the desired bands of the covariate under study, the RIF computes the relevant statistics of each band, as described in the preceding section. Then we can ask the program to build a new index with these levels to standardize rates in future studies. [See Gómez et al. (2002) for computational details.]

In each analysis we performed within the RIF, we can compare results obtained before and after standardization by levels of a covariate. For example, we want to know if calcium concentration in drinking water is a relevant covariate once we have considered the magnesium concentration. Thus, we have compared bands defined from calcium levels after standardization by levels of magnesium. Heterogeneity of these bands will indicate that calcium provides relevant information beyond that supplied by magnesium. Furthermore, comparison of calcium bands before and after standardizing by levels of magnesium will illustrate the interaction of both factors.

### Disease Mapping

One main objective of epidemiologic surveillance tasks is the detection of regions that have unusually high risk. Disease mapping is a powerful tool designed to this end, especially when we are dealing with environmental risk factors. Because environmental phenomena are linked to geography, the influence of these risk factors can be detected by geographic representations of relative risks. [See Lawson and Williams (2001) for an introductory text and Lawson et al. (1999) for a deeper insight.]

Disease mapping deals typically with small geographic units. If the influence of hidden environmental factors extends over several units, mortality/morbidity counts will be correlated. Therefore, to analyze these units we need statistical models allowing for spatial correlation.

Furthermore, the small populations attached to these geographic units produce unstable estimates of relative risks, thus requiring more robust statistical methods.

The RIF addresses both problems by resorting to the empirical Bayes analysis of a hierarchical Poisson-gamma model similar to that of Clayton and Kaldor (1987). Computational details are described in the statistical appendix of Aylin et al. (1999).

From a surveillance perspective, we want to determine if removing the effects of a covariate changes the geographical pattern of relative risks. To this end we can perform disease mapping before and after standardization by levels of a covariate. By comparing the resulting maps, we can verify whether high-risk regions move to lower levels of risk or if they remain high, indicating that factors other than this covariate are still affecting population health status. There could be hidden factors not included in the study. The geographic pattern can help us determine the nature of these hidden factors.

## Results

To delimit the size of the studied phenomena, we first considered the annual rates per 100,000 inhabitants for the whole region. The annual rates for cerebrovascular disease from 1991 to 994 are 153.37 for women and 114.26 for men. From 1995 to 1998 these rates are 129.03 and 97.17, respectively.

The annual rates for ischemic heart disease per 100,000 inhabitants are 80.13 for women and 121.46 for men from 1991 to 1994, whereas they are 86.41 and 126.10, respectively, from 1995 to 1998.

Cerebrovascular disease rates are higher in women than in men; for ischemic heart disease the rates are higher in men. Comparing both periods, we observe a decrease in the rate of cerebrovascular disease and an increase in the rate of ischemic heart disease.

The subsequent analysis focuses on relative risks rather than on the rates and is based on the routine output of the RIF.

### Exposure Analysis

**Figure 1:**
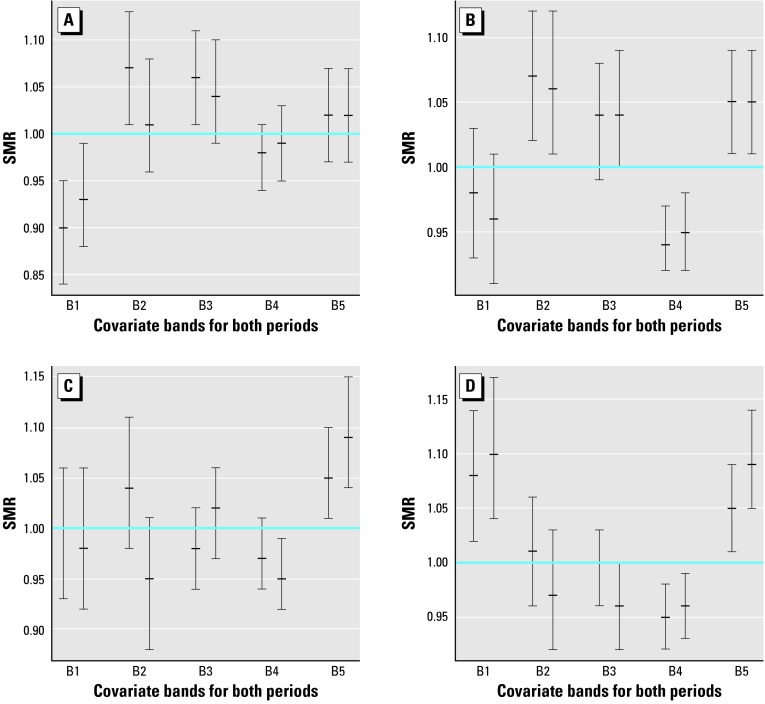
95% Confidence intervals and means of SMRs for cerebrovascular mortality of males (*A,C*) and females (*B,D)* in bands defined for calcium (*A,B*) and magnesium (*C,D*). B, band.

**Figure 2:**
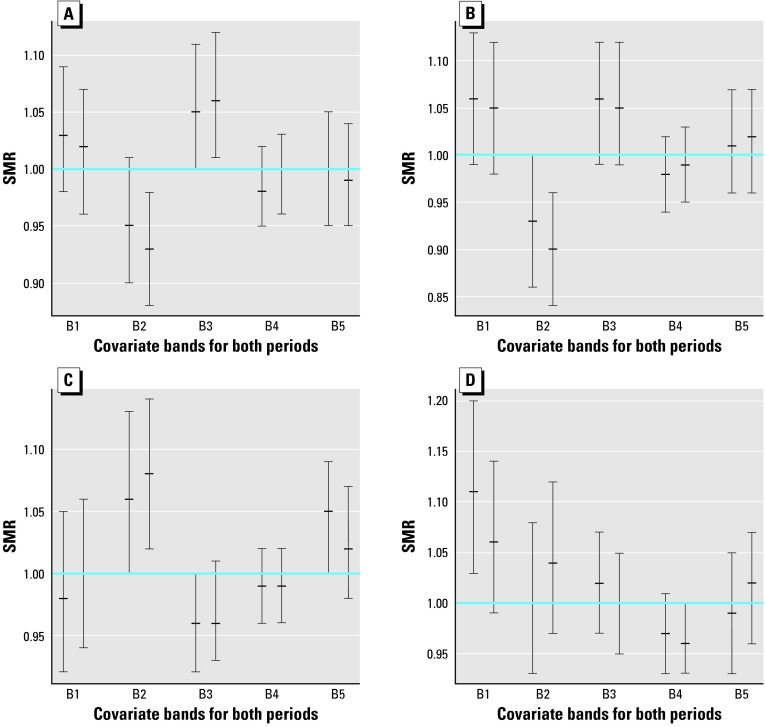
95% Confidence intervals and means of SMRs for ischemic heart mortality of males (*A,C*) and females (*B,D*) in bands defined for calcium (*A,B*) and magnesium (*C,D*). B, band.

Figures 1 and 2 are a comparison of the relative risk of bands defined from calcium and magnesium concentration levels. They display the SMRs and the 95% confidence intervals from the output obtained with the RIF. Figure 1 illustrates cerebrovascular mortality and Figure 2 illustrates ischemic heart mortality. All SMRs for these figures have been computed after standardization by age and deprivation index.

For each disease we have constructed four plots according to sex and covariate. In each band, both 1991–1994 and 1995–1998 are represented side by side for better comparison of temporal variation.

The horizontal line at SMR = 1 allows quick recognition of those intervals not containing this particular value, that is, those intervals that were not significant because the corresponding band shows a significantly high or low relative risk. Because we have performed intrinsic indirect standardization, distance from the SMR = 1 indicates a difference with respect to the average behavior of the whole region. The presence of significant confidence intervals is a clear sign of the heterogeneity of the bands.

As we discussed in “Material and Methods,” this information has to be complemented with testing the homogeneity of the bands. The resulting chi-square statistic G and corresponding *p-*values are displayed in Table 2 under the headings G and *p*-value.

### Magnesium after Calcium and Calcium after Magnesium

**Figure 3:**
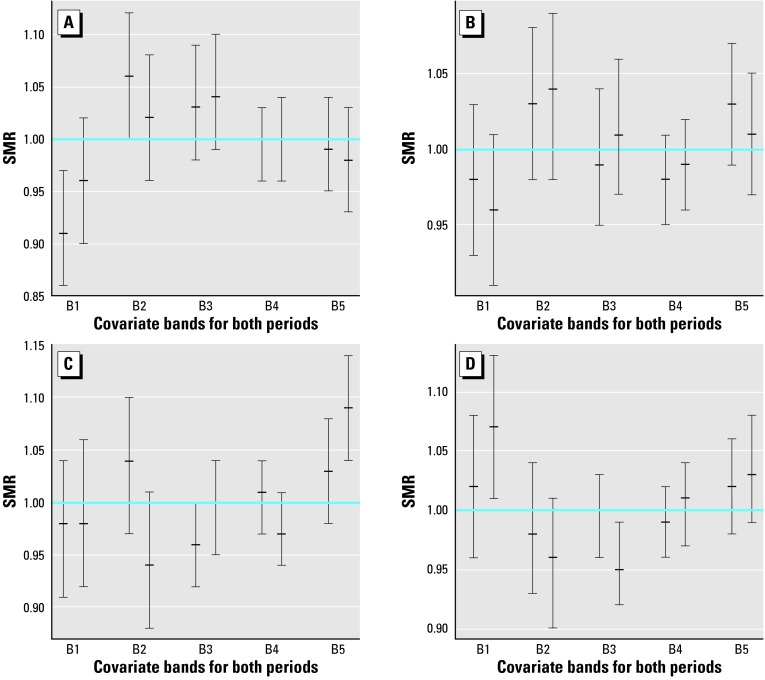
95% Confidence intervals and means of SMRs for cerebrovascular mortality of males (*A,C*) and females (*B,D*) in bands defined from calcium (*A,B*) and magnesium (*C,D*). B, band.

**Figure 4:**
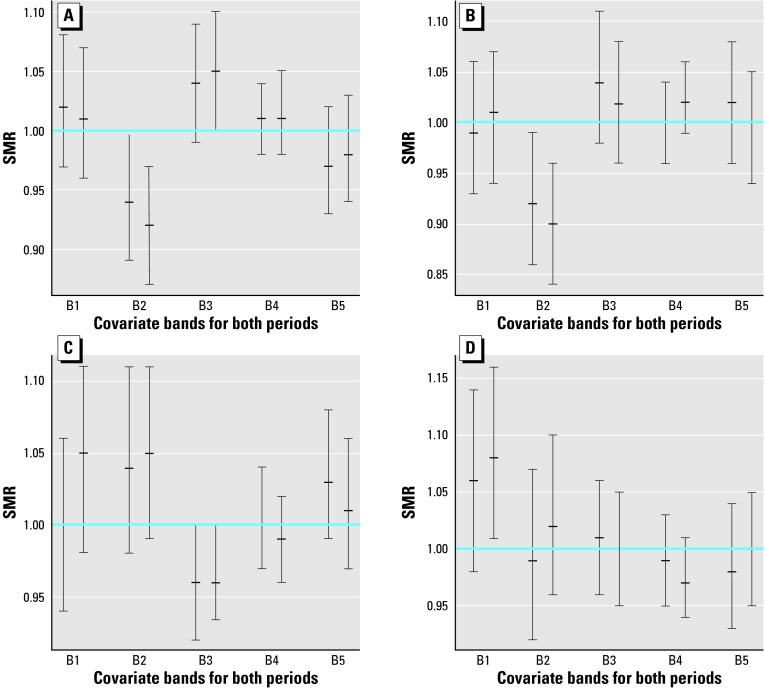
95% Confidence intervals and means of SMRs for ischemic heart mortality of males (A,C) and females (*B,D*) in bands defined for calcium (*A,B*) and magnesium (*C,D*), after standardization by calcium *C,D*) and magnesium (*A,B*). B, band.

To see the additional effect of each covariate once the other has been taken into account, we have repeated the analysis of the preceding section. This time, however, SMRs have been computed after standardization by the covariate not explicitly present in the exposure analysis. Consequently, Figure 3 has to be compared with Figure 1 and Figure 4 with Figure 2. The corresponding homogeneity tests appear in Table 2 under the headings G and *p-*value.

From these comparisons we can see that trends are similar in general but that confidence intervals become less significant. Many more intervals intersect the horizontal line SMR = 1 when we standardize by the covariate not present in the exposure analysis. This loss of significance is apparent as well from columns *p*-value and *p*-value of Table 2. We verify there that *p*-values increase notably from from first column to second column, indicating that the hidden covariate contributes to the heterogeneity between bands. Conversely, small *p-*values suggest the covariate that defines the bands still provides useful information beyond that of the covariate used in standardization.

### Disease Mapping

Rapid Inquiry Facility output gives tabulated SMRs for all municipalities. Shown for each sex group (males, females, and males + females) for each municipality are observed and expected number of cases, the corresponding SMR and the 95% confidence interval, and the smoothed estimation of this SMR based on the empirical Bayes procedure mentioned in preceding sections. These rows are duplicated to show standardized and nonstandardized results.

Because we are working with 538 municipalities and two diseases, the textual output is more than 500 pages for each of the studied covariates. Although interesting for detailed consultation purposes, it does not fit in the reduced space of a scientific paper. Maps better summarize these results. They facilitate the capture of essential aspects of health status. However, the entire set of maps for the present study is excessive, and we will restrict ourselves to some of the most illustrative results.

**Figure 5:**
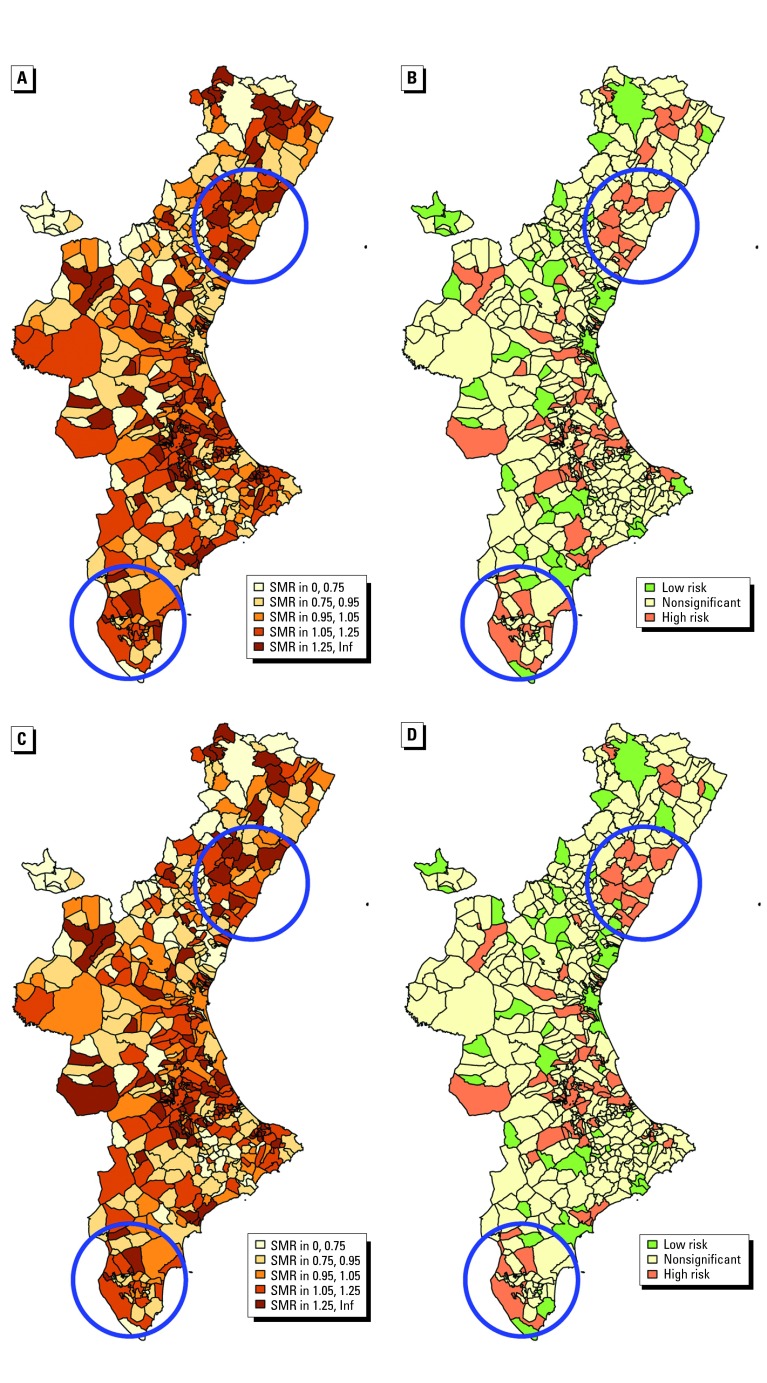
Disease mapping of total cerebrovascular mortality for the whole period: smoothed SMRs (*A,C*) and significance of 95% confidence intervals (*B,D*) after standardization by age, sex, and deprivation index (*A,B*) and further standardization by Mg (*C,D*). Inf, infinity. Municipalities illustrating the change of risk level when adjusting for the covariate are circled in blue.

Figure 5 presents disease mapping of total (males + females) cerebrovascular mortality for 1991–1998. Figure 5A and C represent smoothed municipal relative risks. Figure 5B and D distinguish between significantly high, significantly low, and nonsignificant 95% confidence intervals of SMRs, as the value SMR = 1 is below, above, or inside the interval. Thus, we have an estimate of the relative risk (Figure 5A,C) jointly with a measure of our confidence that the value represents a real risk and is not being produced by mere chance (Figure 5B,D).

In Figure 5A and B, SMRs have been standardized by age, sex, and deprivation index. In Figure 5C and D, standardization has included magnesium levels as well.

## Discussion

### Effects of Calcium and Magnesium

The results displayed in Table 2 suggest a relationship between calcium and magnesium and the data on mortality from cerebrovascular and ischemic diseases. According to the *p*-value, testing homogeneity of bands shows clear evidence of this association for cerebrovascular disease in women. All *p*-values are below 0.0001 for both periods studied and both covariates. Evidence of this association is not as strong for this same disease in men, because there is no clear significant result from 1991 to 1994 with magnesium and from 1995 to 1998 with calcium.

For ischemic heart disease significant heterogeneous results are not achieved if the threshold is set to 0.001 to declare a *p*-value significant. Nevertheless, the *p*-values are quite small, with most between 0.001 and 0.05. A cautious conclusion could be not to discard the possibility of this association without further consideration.

We can examine the nature of those relationships. Focusing on the plot for women and magnesium in Figure 1D (*p*-value = 1.12 × 10^–7^), the one most significant is in Table 2, where we can see a descending trend with increasing levels of magnesium from bands 1 to 4. Band 5 breaks this trend, giving a U-shaped aspect to this plot.

A similar pattern can be seen for ischemic heart mortality in women and magnesium levels, although in this case heterogeneity has not been so significant (*p*-value = 0.031).

Regarding the interaction between calcium and magnesium, Table 2 reveals clearly how the effects of both covariates are partially confounded because they produce in each other a loss of significance when used in the previous standardization. The correlation coefficient for both covariates is 0.59. In this situation it is difficult to assess the independent effect of each one, and it is best to refer to the effect of hardness of drinking water.

In summary, we can say that this study provides statistical evidence of a relationship between mortality from cardiovascular diseases and hardness of drinking water. This relationship is stronger in cerebrovascular disease than in ischemic heart disease, is more pronounced in women than in men, and is more apparent with magnesium than with calcium. Nevertheless, the protective nature of these two factors is not clearly established. Although the results obtained suggest this possibility, they are not conclusive because of the irregular trend in the series of confidence intervals and because many of the results are not significant. Hidden socioeconomic and environmental factors not controlled with the deprivation index or the studied covariates may remain. As suggested by a reviewer, these could be caused by an ecologic bias associated with this region.

### Temporal Trend

We have paired the confidence intervals corresponding to 1991–1994 and 1995–1998 for each band in every plot in Figures 1–4. Direct inspection of these charts reveals the stability of the SMRs during the entire period studied. The majority of these pairs have a large intersection, with both intervals sharing a large portion of their range of values.

Although one may have the impression that small decreases predominate in these sets of paired intervals, the overlapping areas are so important that the evidence of temporal variation is negligible.

### Spatial Distribution of Risk

No spatial trend is apparent from maps presented in Figure 5, but several clusters of different sizes are scattered over the entire region. For illustrative purposes we focused on two particular regions, which are circled in the figure.

The upper circle shows a cluster of municipalities with high SMR and significant confidence intervals. This is obvious in the map of smoothed SMRs standardized by age, sex, and deprivation index (Figure 5A) and in the map with significance of confidence intervals (Figure 5B). The lower circle shows another less extreme cluster.

When we include magnesium levels in the standardization calculus, we remove the effect of this covariate in some sense. When we compare Figure 5A and B with Figure 5C and D, we can see the effect of this removal. For example, the upper circle shows that this change produces even more municipalities with significant confidence intervals than before (so that the situation is worse than previously thought). In the lower circles, the opposite is true. Some municipalities decrease the significance of their SMRs (so that their previous high relative risk has been partially explained by their level of magnesium).

### Some Methodological Issues

The strategy of analysis followed in this study conforms to the exploratory nature of the RIF as a tool to get quick and flexible insight into epidemiologic surveillance problems.

One primary concern with the type of exposure analysis described here is the sensitivity of results to the number and cut points of exposure bands. An advisable practice is to try various configurations of these bands. We still lack a clear recommendation about this topic.

**Figure 6:**
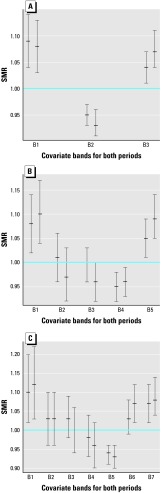
Comparing settings of magnesium bands for cerebrovascular mortality in women. Output obtained with (*A*) three bands, (*B*) five bands, and (*C*) seven bands. B, band.

In our study we used three, five, and seven bands in each of the 12 studies (disease–covariate–sex combinations). Table 3 shows results obtained in one of these comparisons—cerebrovascular mortality in women with bands defined from magnesium levels. Figure 6 displays the SMRs and their 95% confidence intervals from the output obtained with three, five, and seven bands. We can see that although concrete numerical results vary, the general conclusions remain.

Considering these 12 comparisons of different band settings, we found that three bands tend to produce less significant heterogeneous results than with five and seven bands, whereas there is little difference between these last settings. Therefore, we have presented results with five bands.

## Conclusions

Water in the Comunidad Valenciana is very hard. Because of the infrequent occurrence, water with > 200 mg/L calcium and water containing > 10 mg/L magnesium are considered rare by the World Health Organization (1996). Nearly 20% of Comunidad Valenciana municipalities and population have water supplies with calcium concentrations > 200 mg/L. The case of magnesium is more striking—90% of the water supply contains magnesium concentrations > 10 mg/L. This could make identification of dose–response patterns and comparison between our results and those from other studies difficult. Nevertheless, we observed considerable consistency in a detailed analysis of the results obtained in the most recent studies of this issue.

In other studies of calcium, the rank of calcium concentration distribution in water is closer than in our study. For example, in the case–control study performed in Sweden (Rubenowitz et al. 1996), the interval is 22–225 mg/L, and in a recent ecologic study conducted in France (Marque et al. 2003), the calcium concentration interval is 9–146 mg/L. As in our study, mortality results for cardiovascular noncerebrovascular diseases are less clear than those for cerebrovascular diseases. Conversely, the case–control study performed in Sweden shows a nonmonotonic U-shaped relationship between calcium and heart attack risk mortality, as the authors obtained odds ratio < 1 in intermediate calcium concentrations between 34 and 81 mg/L (Rubenowitz et al. 1996). In other words, they found a relationship between heart attack risk mortality calcium levels that correspond to the second level of our distribution, in which we found a significant relationship, with SMR < 1 at concentrations between 65 and 89 mg/L. In a later study in Sweden, the results for calcium were not conclusive (Rubenowitz et al. 2000). Nevertheless, in the French study, the shape of the relationship between calcium and cardiovascular mortality presents a clear biologic gradient, with less mortality risk at higher calcium concentrations in water (Marque et al. 2003).

On the other hand, our results support the protective effect assumption of magnesium and the mortality risk due to cardiovascular diseases (Rubenowitz et al. 1996, 2000). In addition, the results of the study performed in France (Marque et al. 2003) show a U-shaped relation between magnesium concentration in drinking water and cerebrovascular mortality, with lower risk in intermediate values of magnesium, in the same way described in our study.

Briefly, the results of our study in Valencia support the assumption of association between magnesium and mortality risk due to cardiovascular diseases. However, results for calcium are less clear. The current lack of studies and the ecologic nature and limitations of the exposure valuation used suggest that these study results should be explored further with more suitable designs. This could be achieved in the moving cohorts studies framework that addresses the role of different nutrients and other factors in cardiovascular health.

## Figures and Tables

**Table 1:** Bands defined in terms of calcium and magnesium concentrations.

Covariate	Band threshold (mg/L)	Number of muncipalities	Percentage of population
Calcium	[12, 65]	109	13.00
	]65, 89]	110	12.89
	]89, 112]	128	14.93
	]112, 136]	84	39.02
	]136, 480]	107	20.15
Magnesium	[1, 14]	120	8.23
	]14, 23]	96	9.78
	]23, 34]	129	25.00
	]34, 43]	87	38.01
	]43, 117]	106	18.94

**Table 2:** Testing homogeneity of bands.

Disease	Covariate	Period	Sex	G^a^	*p*-Value*^a^*	G*^b^*	*p*-Value*^b^*
Cerebrovascular	Calcium	1991–1994	Males	24.38	6.70 × 10^–5^	14.88	0.0049
			Females	30.75	3.44 × 10^–6^	5.46	0.2436
		1995–1998	Males	8.02	9.06 × 10^–2^	4.85	0.3028
			Females	27.07	1.92 × 10^–5^	4.87	0.3008
	Magnesium	1991–1994	Males	10.16	3.79 × 10^–2^	6.87	0.1427
			Females	21.65	2.35 × 10^–4^	1.71	0.7883
		1995–1998	Males	22.89	1.33 × 10^–4^	16.46	0.0025
			Females	38.00	1.12 × 10^–7^	15.78	0.0033
Ischemic	Calcium	1991–1994	Males	8.85	6.49 × 10^–2^	8.58	0.0725
			Females	11.73	1.95 × 10^–2^	6.77	0.1488
		1995–1998	Males	13.28	9.98 × 10^–3^	14.76	0.0052
			Females	15.14	4.49 × 10^–3^	10.98	0.0268
	Magnesium	1991–1994	Males	10.75	2.96 × 10^–2^	7.16	0.1275
			Females	10.64	3.10 × 10^–2^	2.80	0.5918
		1995–1998	Males	10.62	3.11 × 10^–2^	8.63	0.0710
			Females	8.02	9.08 × 10^–2^	6.98	0.1368

G, chi-square statistic defined by Equation 1.

**a**After standardization by age and deprivation index.

**b**After further standardization by levels of the other covariate.

**Table 3:** Testing homogeneity of bands for cerebrovascular mortality in women with 3, 5, and 7 magnesium bands.

Bands (*n*)	Period	G	*p*-Value
3	1991–1994	34.830	2.74 × 10^–8^
3	1995–1998	52.580	3.82 × 10^–12^
5	1991–1994	21.650	2.35 × 10^–4^
5	1995–1998	38.000	1.12 × 10^–7^
7	1991–1994	38.950	7.32 × 10^–7^
7	1995–1998	49.920	4.88 × 10^–9^

G, chi-square statistic defined by Equation 1.
